# Integrating a community-based continuous mass dog vaccination delivery strategy into the veterinary system of Tanzania: A process evaluation using normalization process theory

**DOI:** 10.1016/j.onehlt.2023.100575

**Published:** 2023-06-03

**Authors:** Christian Tetteh Duamor, Katie Hampson, Felix Lankester, Ahmed Lugelo, Joel Changalucha, Kennedy Selestin Lushasi, Anna Czupryna, Emmanuel Mpolya, Katharina Kreppel, Sarah Cleaveland, Sally Wyke

**Affiliations:** a Department of Global Health and Biomedical Sciences, Nelson Mandela African Institute of Science and Technology, Arusha, Tanzania; b Environmental Health and Ecological Sciences Thematic Group, Ifakara Health Institute – Tanzania, Ifakara, Tanzania; c Boyd Orr Centre for Population and Ecosystem Health, Institute of Biodiversity, Animal Health & Comparative Medicine, College of Medical, Veterinary and Life Sciences, University of Glasgow, Glasgow, UK; d Paul G. Allen School for Global Health, Washington State University, Pullman, WA, USA; e Global Animal Health Tanzania, Arusha, Tanzania; f Sokoine University of Agriculture, Morogoro, Tanzania; g Department of Public Health, Institute of Tropical Medicine, Antwerp, Belgium; h Institute of Health and Wellbeing, College of Social Sciences, University of Glasgow, Glasgow, UK

**Keywords:** Rabies, Process evaluation, Normalization process theory, Community-based, One Health, Vaccination coverage, Mass dog vaccination

## Abstract

**Abstract:**

Sustained vaccination coverage of domestic dog populations can interrupt rabies transmission. However, challenges remain including low dog owner participation, high operational costs associated with current (centralized and annually delivered (pulse)) approaches and high dog population turnover. To address these challenges an alternative (community-based continuous mass dog vaccination (CBC-MDV)) approach was designed. We investigated the potential for successful normalization of CBC-MDV into routine practice within the context of local communities and the veterinary system of Tanzania

**Methods:**

In a process evaluation of a pilot implementation of CBC-MDV, we conducted in-depth interviews with implementers and community leaders (*n* = 24), focus group discussion with implementers and community members (*n* = 12), and non-participant observation (*n* = 157 h) of delivery of the intervention components. We analyzed these data thematically drawing on the normalization process theory, to assess factors affecting implementation and integration.

**Main findings:**

Implementers and community members clearly understood the values and benefits of the CBC-MDV, regarding it as an improvement over the pulse strategy. They had a clear understanding of what was required to enact CBC-MDV and considered their own involvement to be legitimate. The approach fitted well into routine schedules of implementers and the context (infrastructure, skill sets and policy). Implementers and community members positively appraised CBC-MDV in terms of its perceived impact on rabies and recommended its use across the country. Implementers and community members further believed that vaccinating dogs free of charge was critical and made community mobilization easier. However, providing feedback to communities and involving them in evaluating outcomes of vaccination campaigns were reported to have not been done. Local politics was cited as a barrier to collaboration between implementers and community leaders.

**Conclusion:**

This work suggests that CBC-MDV has the potential to be integrated and sustained in the context of Tanzania. Involving communities in design, delivery and monitoring of CBC-MDV activities could contribute to improving and sustaining its outcomes.

## 1 Introduction

Annually, rabies is responsible for approximately 59,000 human deaths globally (including $8.6 billion in economic losses) [1] and 552 in Tanzania [2]. Evidence suggests that sustaining vaccination coverage of domestic dog populations above 40% all year round interrupts transmission, but where campaigns are organized only once a year, they must reach at least 70% coverage to ensure herd immunity is maintained [[3], [4], [5], [6]]. The strategy that is mostly used to reach and vaccinate dogs in rabies-endemic countries involves teams of vaccinators traveling annually to communities where temporary mass dog vaccination (MDV) clinics are set up in central locations to which dog owners bring their dogs, to be inoculated with cold-chain stored vaccines. However, this strategy, hereafter referred to as the pulse approach, does not always result in a sustained vaccination coverage above this 40% minimum threshold target throughout the year. Reasons for this include: low dog owner participation, especially if there is insufficient mobilization; features of dog demography in endemic countries, which typically include a high dog population turnover (and hence a rapid decline in coverage after pulse campaigns) [6,7]; and the lack of commitment to government funding to cover the operational costs needed for large-scale MDV [[8], [9], [10]].

We developed a community-based continuous mass dog vaccination (CBC-MDV) approach that aims to provide continuous (all-year-round) access to dog vaccination for communities in rural Tanzania. The approach involves thermotolerant vaccines [11] being stored locally in passive cooling devices [12] and being used in four rounds of MDV campaigns every year, with each round spanning a few days to several weeks, plus additional vaccination being available throughout the year in response to requests by dog owners. We hypothesize that, if successfully implemented, this approach could create and sustain the herd immunity required to interrupt rabies virus transmission [[3], [4], [5],^13^,^14^].

Prior to implementation of a large-scale randomized controlled trial (RCT), we carried out a pilot study embedded with process evaluation from July 2019 to June 2020 to study feasibility of delivering CBC-MDV and how it compares with the pulse approach. We found that one month after the first vaccination campaign, coverage in areas receiving CBC-MDV was higher compared to areas receiving vaccination through the pulse approach. Follow-up surveys 10 months later showed that vaccination coverage in areas receiving CBC-MDV remained considerably higher than in areas receiving vaccination through the pulse approach [13]. We also found that although fidelity of delivery was not perfect, and was influenced by strategy design, implementer availability and local environmental and socioeconomic events (e.g. elections, auctions, funerals, school cycles), it was feasible to deliver the CBC-MDV approach with good vaccination coverage of dogs [14].

In this paper we present an investigation of the potential for successful normalization of CBC-MDV into routine vaccination practice within the context of local communities and the veterinary system of Tanzania using the Normalization Process Theory (NPT). Normalization refers to the process through which an intervention becomes integrated and sustained in practice as the new standard [[15], [16], [17]]. We expect that the results will inform wider implementation of CBC-MDV if the full RCT suggests it is (cost)-effective.

Normalization of new interventions has been shown to be influenced by many factors including: the level of involvement of intervention managers and end users in design and delivery; whether implementers have good knowledge of the intervention and the skill sets to deliver it; organizational support and resources needed for delivery [[18], [19], [20]]; interpersonal relationships among key stakeholders; and clear communication of intervention values and benefits [21].

NPT employs four constructs to describe determinants of routinization of new complex interventions into practice. These are: i) Coherence, implementers' understanding of the new intervention; ii) Cognitive participation, implementers' willingness to engage with it; iii) Collective action, implementers' ability to deliver it collectively including having sufficient resources for delivery; and iv) Reflexive monitoring, implementers' ability to appraise and amend the intervention during the course of implementation [17]. Field notes and interviews conducted during the implementation of the process evaluation, carried out in parallel to the pilot study were explored with the NPT constructs to examine the potential for normalization of CBC-MDV.

## 2 Methods

### 2.1 Study design

Our process evaluation followed the pilot delivery processes of CBC-MDV for a year and qualitatively explored the feasibility of integrating and sustaining it in practice as the new standard approach for delivering MDV in Tanzania. The data were analyzed within the framework of the NPT constructs [15,16] (Table 1).

**Table 1:** Questions associated with the constructs of Normalization Process Theory.

Coherence (Understanding of the new intervention)	Cognitive participation (Willingness to engage with it)	Collective action (Ability and resources to deliver it)	Reflexive monitoring (Ability to appraise and amend)
Differentiation Do stakeholders see a difference between new vs current approach?	**Enrollment** Are stakeholders willing to invest time and energy into it?	**Skill set workability** Are implementers able to deliver the new approach?	**Reconfiguration** Can stakeholders amend the new approach, based on experience?
Communal Specification Is there shared understanding of aims, objectives and benefits of the new approach?	**Activation** Are stakeholders able to define activities and work needed to sustain the new approach?	**Contextual integration** Is the new approach supported by local policy and resources?	**Communal appraisal** Can stakeholders collectively assess effectiveness and benefits of the new approach?
Individual Specification Are individual tasks and responsibilities understood?	**Initiation** Are stakeholders willing and able to get others involved?	**Interactional workability** Does the new approach make completion of routine tasks easy?	**Individual appraisal** Can implementers assess impact of the new approach on them and their roles?
Internalization Are the values, benefits and importance of the new approach appreciated?	**Legitimation** Do stakeholders believe that they should be involved in delivery of the new approach?	**Relational integration** Do stakeholders trust the new approach and implementers?	**Systematization** Can stakeholders judge effectiveness and/or success of the new approach?

### 2.2 Setting

**Fig. 1:**
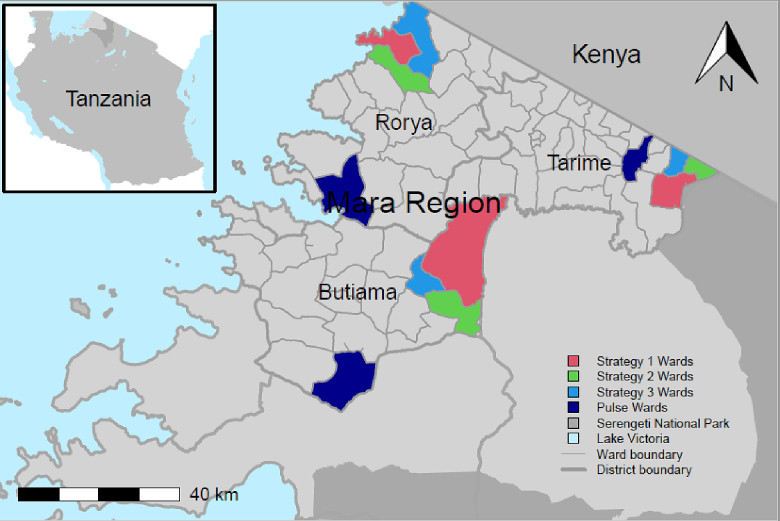
Map of study site showing Mara region and wards involved in the pilot study.

CBC-MDV was piloted in three rural districts of northern Tanzania where rabies remains endemic. The study was conducted at three levels: i) district (*n* = 3) – where logistics for CBC-MDV were managed and vaccination campaigns were supervised by district livestock field officers; ii) ward (*n* = 12) (clusters of 3–4 villages) – where vaccination campaigns were organized by ward livestock field officers; and iii) village (*n* = 35) – where vaccination campaigns were delivered and supported by community members. The districts were purposively selected to ensure equal geographic (level of urbanization and economic activities) and sociocultural (dog ownership practices) representation of the Mara region. A ward from each district was then assigned at random to deliver MDV using one of three strategies of CBC-MDV, while one ward from each district used the pulse approach (Fig. 1). However, the experiences of implementers and communities with the pulse approach expressed here go beyond the pilot study.

### 2.3 Description of CBC-MDV

In contrast to the pulse approach, where MDV campaigns are conducted only once a year, the CBC-MDV was designed to provide continuous access to dog vaccination to communities across these rural and remote landscapes (Additional file). CBC-MDV was developed by the research team with participation of district, regional and national level veterinary and human health, local government, One Health Unit and WHO officials in Tanzania, through five iterative development workshops which took place between May 2018 and May 2019 [14].

CBC-MDV has 45 components [14] which can be categorized into eight key ingredients including:

i) local delivery led by district-level veterinary authorities to foster buy-in. Their primary roles were to receive vaccination materials (vaccines, syringes, needles, certificates, dog muzzles, cotton, spirit, waste bins, cooling boxes and registers) from the research team, distribute the materials to vaccinators at ward levels, introduce vaccinators to their communities and supervise vaccination activities;
ii) involvement of community leadership in mobilizing dog owners;
iii) use of village-based personnel called One Health Champions (OHCs), trained by the research team to support ward-level livestock field officers (called Rabies Coordinators – RCs in this study) in organizing vaccination activities;
iv) widespread communication at village levels about CBC-MDV and advertising of campaigns using multiple channels;
v) use of passive cooling devices [12] to store thermotolerant rabies vaccines in wards to support year-round campaigns;
vi) quarterly delivery of MDV and on-demand vaccination (if requested by dog owners) to sustain herd immunity all year round;
vii) free of charge vaccination to encourage dog-owner participation;
viii) monitoring and feedback on vaccination coverage to district veterinary authorities, vaccinators and communities to reconfigure CBC-MDV where necessary in the course of implementation.

To allow investigation of different ways CBC-MDV can be delivered, the delivery was stratified into three strategies: 1) central point clinics at village level; 2) central point clinics at subvillage level, and 3) delivery approaches chosen at the discretion of implementers. In all strategies, CBC-MDV was delivered on a quarterly basis and, in addition, dog owners were allowed to request that their dogs be vaccinated on an ad hoc basis at any point during the year (on demand).

A manual on CBC-MDV was developed by the research team to guide the implementation process. The district-, ward- and village-level implementers were trained in a 3-day workshop, focusing on the concept of CBC-MDV and skills for its components, with practical sessions delivered in a selected village.

### 2.4 Data collection and participants

To examine the potential for normalization of CBC-MDV, we conducted 24 in-depth interviews with district livestock field officers (DLFOs) (*n* = 3), RCs (*n* = 7), OHCs (*n* = 8) and community leaders (*n* = 6). Whilst every DLFO and RC was interviewed, OHCs and community leaders were purposively selected considering representation across all CBC-MDV wards. We also conducted focus group discussions (FGDs) with RCs and OHCs (*n* = 3, including 9 RCs and 35 OHCs), community leaders (*n* = 3, including 35 leaders), and community members (*n* = 6, including 24 men and 29 women with a wide range of ages). Participants in the community-member FGDs were purposively selected with a view to ensuring representation of leadership, age groups and gender across the 35 villages.

The interviews and FGDs were conducted between December 2019 and July 2020. Both used topic guides (based on the NPT constructs) and were conducted in Swahili, by an experienced interviewer, in-person and after consent was obtained. The interviews lasted between 18 and 65 min and were recorded with an Olympus VN-541PC voice recorder.

We conducted non-participant observations (NPO) (*n* = 172 h) using a structured proforma guide. The NPOs focused on the delivery of advertising and vaccination clinics. The proforma queried: i) advertising methods/ activities; ii) advertising timing and message content; iii) reactions of villagers to advertising; iv) what influenced timing of and turn out at vaccination clinics; vi) fidelity to protocol; (vii) if implementers were able to deliver the CBC-MDV components and vii) enhancers and barriers that they faced.

The topic guides and proforma were revised after the first three interviews and observations respectively.

### 2.5 Data management and analysis

Data from interviews and FGDs were transcribed verbatim, and together with field notes from NPOs were translated into English Language. The transcripts were then assigned unique identifiers and imported into NVivo 12 Plus version 20.5.1.940 [22].

Data were coded by the first author (CTD), guided by a coding manual developed by CTD and the last author (SW), following the 7-stage framework method proposed by Gale et al. [23], and with codes based on the 16 NPT constructs [23,24]. CTD and SW independently applied the coding manual to five transcripts. They then met repeatedly to clarify coding differences until a consensus was reached and the coding frame finalized. All transcripts were then read and assigned codes. The coded extracts were summarized within the 16 NPT constructs (Table 2) to explore feasibility of integrating and sustaining CBC-MDV in practice within the context of communities and the veterinary system of Tanzania.

**Table 2:** Overall findings for routinization of CBC-MDV based on NPT constructs.

Coherence (sense making work)	Cognitive participation (relational work)	Collective action (operational work)	Reflexive monitoring (appraisal work)
Differentiation Communities and implementers perceived CBC-MDV to be different from the pulse approach. The differences were that CBC-MDV involved the community in planning, created more awareness, was flexible, continuous, more accessible and reached more dogs including new puppies.	**Enrollment** Community leaders and implementers were prepared to invest time and energy into CBC-MDV: they perceived it as part of their responsibilities; and made time by planning.	**Skill set workability** Community leaders and DLFOs believed implementers have the required skills to deliver CBC-MDV. Non-participant observation noted implementers competently delivered most CBC-MDV components.	**Reconfiguration** Implementers did not feel they had the power to vary the intervention in the course of implementation based on their experience.
Communal Specification Communities and implementers understood the aims, objectives and benefits of CBC-MDV as to achieve the vaccination of more dogs and prevention of rabies and its impacts.	**Activation** Community leaders defined activities and work needed to sustain CBC-MDV as including: mobilization of community members, rabies education, advertisement of dog vaccination, creation of a community register of dogs for monitoring, preparing annual timetables for dog vaccination, enacting by-laws to enforce vaccination; and secure funding. Implementers defined activities as: coordination of activities, creating awareness at village meetings and planning timetables	**Contextual integration** Community leaders believed some by-laws exist and should be enforced to support CBC-MDV: i.e., that people must vaccinate their dogs every year with fines imposed on those who fail and that owners must pay for post-exposure treatment if the unvaccinated dog bites someone. They also believed if communities were involved in planning, CBC-MDV could be included in the community budget Communities and implementers advocated for donor support for vaccines and equipment. Fridge and office spaces were available for keeping vaccination materials at district offices, some village offices provided room space for passive cooling devices and others released tables and chairs for clinics.	**Communal appraisal** Community leaders and implementers collectively assessed effectiveness and benefits of CBC-MDV as: less frequent rabies cases including in livestock; prevention of the costs of human vaccines when a biting dog is vaccinated; the dog vaccination service is more available now and more dogs are vaccinated. However, providing feedback to communities and involving them in evaluating outcomes of vaccination campaigns was not done.
Individual Specification Understanding of individual tasks and responsibilities: Community Leaders understood their responsibilities included mobilization, education and inspiration of people on controlling rabies; to enact by-laws; to supervise, monitor and report vaccination activities DLFOs [..] to enforce government policies on animal diseases control including rabies; to train and supervise implementers. LFOs […] to provide education on rabies, organize and report on campaigns. OHCs […] to educate and mobilize communities, estimate or document the number of dogs needing vaccination, advertise campaigns, registration and certification of vaccinated dogs.	**Initiation** Willingness and ability to involve others: community leaders and implementers appreciated the need to get community-level committees and members involved in CBC-MDV; they stated that they have authority to convene meetings. For example, one DLFO involved the district commissioner and executive director; two wards (strategy 3) decided on their own delivery approach with communities and some OHCs helped with activities in other villages within their wards.	**Interactional workability** Implementers observed they are able to continue with routine tasks because they know the timetable of CBC-MDV for the whole year. CBC-MDV made dog vaccination easier to implement because: it involved communities in planning, vaccines and funds were available, the passive cooling devices ensured easy access to vaccines throughout the year because vaccines were stored in communities.	**Individual appraisal** Community leaders and members said they now understood the advantages of dog vaccination; they don't have to take many injections (PEP) when bitten by a dog. Implementers thought the community was convinced about the importance of dog vaccination and were satisfied with the outputs of CBC-MDV.
Internalization Communities and implementers believed the value, benefits and importance of CBC-MDV included: reaching communities sustainably at reduced cost, allowing local input, involving community-based implementers to improve mobilization, protecting people and dogs from rabies and averting expensive treatment of dog bites.	**Legitimization** Community leaders believed: their involvement helped to make dog owners responsive to the vaccination team and thought, if involved in planning and monitoring of CBC-MDV, implementation would improve Implementers believed it is part of their responsibilities to control rabies, they also considered their involvement as serving their communities.	**Relational integration** **Community leaders and members** said they trusted CBC-MDV and its implementers because of recognition by district veterinary offices, and they did not see any negative impact of the vaccine on dogs **DFLOs** said communities trusted the program and its implementers because the OHCs were selected from the communities and communities had access to the vaccinators.	**Systematization** Communities and implementers agreed that CBC-MDV should be used to deliver dog vaccination across Tanzania because: they believed it reached more dogs, involved the community, fostered ownership of dog vaccination, improved awareness of rabies, was user friendly, available most of the time and sustainable.

## 3 Results

### 3.1 Findings with implications for normalization through the NPT constructs

Key findings for likelihood of integrating and sustaining CBC-MDV in practice are summarized under the 16 NPT constructs in Table 2.

### 3.2 Coherence – making sense of CBC-MDV

Implementers, community leaders and members understood the aims, values and advantages of the CBC-MDV strategies: i) they perceived CBC-MDV as a more inclusive approach to mobilizing dog owners and they perceived CBC-MDV as providing better access to dog vaccination compared to the pulse; ii) they clearly identified how the two approaches differed; and iii) they understood the tasks ascribed to them:

“*There is a big difference since the community-based strategy involved team work in making an action plan and also involved people from the particular community and so this made it much easier to reach more dogs*” [Community Leader-3 IDI, District 1].

“*My responsibility is to mobilize the community in collaboration with the livestock field officers who are educating people, when they plan to vaccinate, I call the ward development committee to discuss and we give responsibilities to each other and emphasize to the community to bring their dogs*” [Community Leader-1 IDI, District 2].

“*My first role as a veterinary doctor is to prevent livestock diseases, so one of my responsibilities in rabies control is to use appropriate methods to prevent and protect the community and animals from contracting the disease. That is my responsibility and I perform it by providing rabies vaccine*” [DLFO IDI, District 1].

These views illustrate that CBC-MDV made sense to those involved and thus has potential to be integrated into their routines.

### 3.3 Cognitive participation – investing in CBC-MDV

Implementers and communities showed willingness to engage with CBC-MDV: i) they considered their involvement in CBC-MDV to be legitimate and that they should make time for it; ii) they had clear understanding of what was required to enact and sustain CBC-MDV implementation and iii) knew who should be involved to ensure success:

“*I found it easy to make time for CBC-MDV because dog vaccination is among my responsibilities as a livestock field officer, so I was using my normal timetable*” [RC-2 IDI, District 1].

“*I think that the community should be provided with adequate education about rabies, they should be educated on the benefits of vaccinating their dogs. Secondly, laws should be made to hold people accountable … when this (vaccination) exercise is completed we should conduct an inspection from house to house to verify that all dogs have been vaccinated and those who did not vaccinate their dogs intentionally will be made to face the law*” [P 3, Implementers FGD, District 3].

“*The main issue is community mobilization so as to make them aware about the importance of vaccination*” [P 9, Adult Male FGD, District 3].

Communities and implementers also indicated how CBC-MDV can be improved further through extended community participation:

“*We should engage all levels from ward to village to subvillages … to have a vaccination timetable by putting it into our work plan*” [P 9, Community Leaders FGD, District 1].

*“… also using different leaders such as Ward Executive Officers, Village Executive Officers, Village and Subvillage Chairpersons who can advertise easily to the community to bring their dogs for vaccination”* [P 7, Implementers FGD, District 3].

And cited examples of how involvement of community leaders had helped:

“*It was the mobilization done by our community leaders in village meetings, so we had to take our dogs for vaccination*” [P 5, Adult Females FGD, District 2].

Politics was considered a potential barrier to strong community collaboration and participation in CBC-MDV delivery:

“*The major thing is politics, sometimes people involve politics and different opinions but otherwise there is no problem if the community is directly involved*” [DLFO IDI, District 2].

“*What hindered me were political issues, my area is led by the opposition party. So, when we are mobilizing for this exercise others considered it as a strategy for the ruling party to campaign, that was one of the challenges we faced*” [RC-2 IDI, District 2].

These views illustrate awareness of what was required and willingness to enact CBC-MDV into practice.

### 3.4 Collective action – implementing the CBC-MDV protocol

The implementers found it relatively simple to operationalize the CBC-MDV protocol: i) district-level implementers managed logistics efficiently and ward/ village-level implementers delivered CBC-MDV components satisfactorily; ii) infrastructure (fridge and room spaces to keep vaccination materials) and by-laws to support CBC-MDV were available; iii) having an annual schedule for vaccination activities helped implementers in planning their routine tasks; iv) availability of [research fund & local] resources made delivery of CBC-MDV easier; and v) secondment of implementers by DLFOs and community leadership fostered trust in CBC-MDV and its implementers.

“*There are (resources to support CBC-MDV), is in my village government office that I stored the reports and equipment for the vaccination exercise. [...], the chairs and tables I used belong to the respective village government offices*” [RC-1 IDI, District 3].

The support was based on trust:

“*Yes, they (communities) have trust in them (vaccinators), because they come from the same communities*” [DLFO IDI, District 1].

The district veterinary officers trusted in the abilities of vaccinators to deliver CBC-MDV:

“*They have skills because they have studied about these things but also received training from the project, apart from* learning *on their jobs as livestock officers, the project continues to educate them*” [DLFO, IDI, District 1].

These quotes indicate that CBC-MDV can be integrated in the context of Tanzania with relative ease.

### 3.5 Reflexive monitoring – recommending CBC-MDV

While both implementers and communities positively appraised CBC-MDV, sharing of feedback among the research team, implementers and communities was lacking and implementers were not aware upfront that they could vary the CBC-MDV protocol in the course of its implementation; as demonstrated in the following quotes respectively:

“*Yes, and I would like this strategy (CBC-MDV) to be sustained because it reduces rabies, it brings vaccination centers close to even those who are living far*”. [P 5, Mixed Young People FGD, District 1].

*“There must be an evaluation, for example in our zone we expected to vaccinate 1,000 dogs but after implementation, how many dogs have we vaccinated? And if we failed to reach our goal what are the causes? That will help to make plans to improve the next implementation”* [P 3, Community Leaders FGD, District 3].

“*No, we did not consider experience (to modify the protocol), but we considered the level of mobilization and how the community perceives the programme (dog vaccination) and then explained it to them*” [RC-1 IDI, District 2].

Communities also appreciated the potential benefits of CBC-MDV:

“*I just mention one, as a person gets bitten and rushed to hospital, you might find there are no* post-exposure *vaccines. But after this program there will be no high risk, because the dogs were already vaccinated*” [Community Leader-3 IDI, District 2].

Community members suggested ways that implementers can be empowered and how to ensure continuity of dog vaccination including easy access of implementers to the villages, and that vaccination should continue to be free of charge:

“*By modification, I mean vaccination teams should be empowered with transport facilities for easy and early access to vaccination centers. … to motivate personnel so they can go around the village frequently*” [P 8, Community Leaders FGD, District 3].

“*Also, the government should provide vaccines in a sustainable manner*” [P 5, Community Leaders FGD, District 3].

“*Frankly, the major policy which causes the community members to bring their dogs is that the vaccination is free of charge*” [OHC-33 IDI, District 3].

“*As it has been sponsored up to now it is a good thing, for example, vaccines are here and for free. That led to easy community mobilization for mass dog and cat vaccination. But if it would rely on community contribution, I think this would not be possible*” [P 2, Community Leaders FGD, District 3].

These views of what is needed to mobilize communities, and suggestions regarding how CBC-MDV could be improved, were further expressed in the way that implementers think CBC-MDV should be monitored:

“*if each village has a register of dogs, it will be easy to monitor who has brought their dogs for vaccination*” [OHC-23 IDI, District 2].

Some community leaders expressed similar views:

“*Also, they have to provide a register of vaccinated dogs to community leaders. For example, if it is per household, then it will be easy to identify unvaccinated dogs. They have never given us a register after that exercise of dog vaccination in our village although we participated in mobilization*” [P 9, Community Leaders FGD, District 3].

Community views on CBC-MDV campaign strategies were that villages should be divided into zones so that each zone has an OHC and a vaccination center:

“… *setting vaccination centers near communities will help even lazy ones to bring their dogs*” [P 5, Community Leaders FGD, District 3].

“*Maybe I can say it is the large size of this ward, walking to every place to reach the community to educate them about this matter, and most of our people live far in the bush, that is a challenge*” [Community Leader-1 IDI, District 3].

These sentiments have contributed to considerations regarding how normalization of CBC-MDV can be facilitated.

### 3.6 How normalization of CBC-MDV can be facilitated

**Fig. 2:**
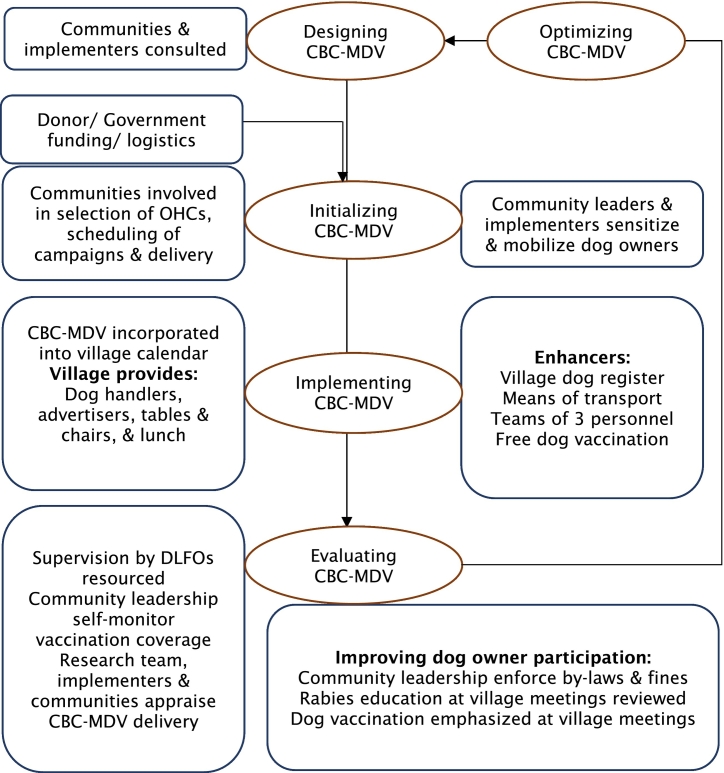
Community and implementer mental model of how CBC-MDV should be designed, delivered and evaluated.

Based on the views of implementers and communities presented in the NPT analysis we constructed a stakeholder mental model of approaches to designing, implementing and evaluating CBC-MDV to facilitate integrating and sustaining it in practice (Fig. 2).

## 4 Discussion

This study used NPT to investigate the potential for successfully integrating and sustaining CBC-MDV in practice within the context of local communities and the veterinary system of Tanzania. Our findings were that: implementers and communities clearly identified the values and benefits of CBC-MDV and considered the approach as being more effective in reaching more dogs compared to the pulse approach (Coherence). They had clear understanding of what was required to implement CBC-MDV, and thought it was legitimate for them to participate and get others involved in the process (Cognitive Participation). Implementers satisfactorily delivered components of CBC-MDV, and had infrastructure to support logistics management, whilst communities identified local policies to support integration of CBC-MDV (Collective Participation). Both implementers and communities positively evaluated CBC-MDV in terms of its impact on rabies cases and implications for post-exposure prophylaxis use and recommended that CBC-MDV be expanded across the country. However, there was lack of routine feedback sharing among the research team, implementers and communities. In addition, implementers did not perceive that they could adapt the CBC-MDV protocol during implementation based on their experience (Reflexive Monitoring), which was considered important in the strategy design. Overall, these findings suggest strong potential for normalization of CBC-MDV, but also areas for improvement that were considered in the ongoing scaled-up RCT.

### 4.1 Coherence

That CBC-MDV made coherent sense to implementers and communities appeared to be due to the training of implementers before rollout and more than 12 months of interaction with the intervention. Having clear understanding of how CBC-MDV was designed to function helped the value judgments of how process activities could lead to expected outcomes and led to clear differentiation of CBC-MDV from the pulse approach. As noted previously, having a good knowledge of CBC-MDV could have fostered acceptance on the part of implementers and communities [25,26]. A broader participation in design of CBC-MDV could further enhance a common understanding of its purpose and elicit stronger willingness to implement it [27,28].

### 4.2 Cognitive participation

Implementers and communities perceived their roles in CBC-MDV as part of their jobs, that fit well into their routines and made it simpler for them to deliver it. How much time implementers have to understand a new practice; regarding how it might impact existing routines, operational tasks and regulations required, and its advantages, influences how an intervention is enacted into practice [29]. These reflections are also important for legitimization and buy-in to a new intervention and are key to successful implementation [[30], [31], [32]]. Despite the acceptance of CBC-MDV by those involved, respondents also considered how its design, implementation and evaluation could be improved with many suggesting it was important for communities to be more directly involved in these processes. Co-design is cited to afford implementers and communities opportunities to contribute towards building an understanding of how a new intervention could work [33,34], with a subsequently increased tendency for adoption and ownership [35,36]. Local politics, cited as a barrier to collaboration between community leaders and implementers in CBC-MDV demonstrates how people with vested political interests might derail or capture collaborative efforts to their advantage [37]. Evidence of this was described in a report of a community-based programme delivering Newcastle Disease vaccine where it was reported that the question of who controlled the resources and power that came with the project strained relationships among community leaders and vaccinators [38].

### 4.3 Collective action

CBC-MDV was operationalized with relative ease because sufficient resources were provided for effective training of implementers and it fitted well into their routines. Additionally, ease of delivery of CBC-MDV was ensured through availability of resources such as space in district and village offices for fridges and storage of vaccination materials and passive cooling devices, respectively, the availability of tables and chairs from village offices for use during clinics and funds from the research project. This suggests that, outside of this project, if funding is secured and the strong community leadership support for CBC-MDV is harnessed, CBC-MDV can be integrated [18] and sustained in practice [35,36] in Tanzania. In contrast to findings of other studies, where implementers resisted new interventions because operational tasks and the realities of the new intervention added complexities or required additional efforts or time to deliver [21,39,40], CBC-MDV was accepted by implementers as it fitted well into their routines and matched their skills. Similarly, in a bone fracture prevention study, it was noted that putting in place designated services coordinators freed up healthcare professionals and enhanced their capacity to enact components of the intervention [20]. The training CBC-MDV implementers received also facilitated its operationalization. As noted by another study, the amount of training implementers are given influences enactment and routine use of a new intervention [18].

### 4.4 Reflexive monitoring

The CBC-MDV implementation manual prescribed that the district veterinary office and the research team will provide feedback on vaccination coverage to communities after each round, and communities to monitor delivery of CBC-MDV; these were not implemented. It is likely that the spread of COVID-19 during the time of the study contributed to this lack of engagement from the research team. But also, feedback activities imposed extra work, which may have deterred implementers from carrying them out. This is similar to findings from an implementation study of a digital patient feedback intervention where the health staff perceived feedback activities as an added burden [41]. The elaborate community leadership structure of Tanzania (a significant administrative connection among ward, village, subvillage, hamlet and household leaderships) provides a good platform to establish village-level monitoring of CBC-MDV delivery. Reflexive monitoring also permits value judgements of an intervention and whether it should be sustained in practice [42]. In this regard, both communities and implementers recommended CBC-MDV be adopted as the standard approach for delivering MDV across the country. This suggests CBC-MDV could be integrated and sustained in practice should large-scale evaluation results support its dissemination nationwide.

### 4.5 Strengths and limitations of this study

The NPT theory provided a strong theoretical basis for this study, and our prospective design afforded us the opportunity to follow the intervention through its development, initialization and implementation phases, reducing recall bias. Given the similarities in many respects of dog ownership practices, socioeconomic and environmental factors between the study area and settings in other low- and middle-income countries especially in Tanzania, the findings may also be transferable. However, the findings will likely be less applicable to more urban areas and nomadic communities which differ considerably in terms of dog ownership and management practices. Additionally, the positive views of implementers, community leaders and community members may be due to their enthusiasm for new programs and could potentially abate unless efforts were put in place for continued engagement. Again, the expressed opinions of community leaders and community members in support of delivery of dog vaccination through the CBC-MDV approach will need to be tested in actual implementation to learn how that works.

## 5 Conclusions

This work suggests that CBC-MDV has the potential to be integrated and sustained in practice in the context of Tanzania. Enabling broad community participation in the design, implementation, evaluation and feedback of CBC-MDV activities could foster improved tailoring of the intervention to local contexts, thereby strengthening community interest in and contributions towards delivery of mass dog vaccination to reduce the burden of this neglected but entirely preventable disease.

## Ethics approval and consent to participate

The protocol for this study was reviewed and approved by the Institutional Animal Care and Use Committee, Washington State University [Approval No. 04577 – 001], the Tanzania National Medical Research Institute [NIMR/HQ/R.8a/Vol.IX/2788], the Tanzania Regional Administration and Local Government [AH.213/420/01] and the Ifakara Health Institute [IHI/IRB/No:024-2018]. Administrative permissions were sought from Rorya, Butiama and Tarime district veterinary offices and the leaderships of the wards and villages involved in the study. Participants received information aim and procedures of the study, they were then allowed time to ask question and agreed to participant by signing a consent form.

## Consent for publication

Participants made aware during the consenting process that their views will be shared widely including publication in peer review journals.

## Funding

Funding for the postgraduate study of (CTD) and supervision by (EM & KK) was received from the DELTAS Africa Initiative [Afrique One-ASPIRE/DEL-15-008]. Afrique One-ASPIRE is funded by a consortium of donors, including the 10.13039/501100011858African Academy of Sciences (AAS), 10.13039/501100014163Alliance for Accelerating Excellence in Science in Africa (AESA), the New Partnership for Africa's Development Planning and Coordinating (NEPAD) Agency, the Wellcome Trust [107753/A/15/Z] and the 10.13039/100013986UK government.

The mass dog vaccination and research activities were funded by the Department of Health and Human Services of the 10.13039/100000002National Institutes of Health [R01AI141712]. The content is solely the responsibility of the authors and does not necessarily represent the official views of the National Institutes of Health.

Dog vaccines for mass dog vaccination were donated by MSD Animal Health.

The 10.13039/100010269Wellcome Trust funded KH and CTD [207569/Z/17/Z].

None of the funders had a role in study design, data collection and analysis, decision to publish, or preparation of the manuscript.

## CRediT authorship contribution statement

**Christian Tetteh Duamor:** Conceptualization, Data curation, Formal analysis, Investigation, Methodology, Project administration, Validation, Visualization, Writing – original draft, Writing – review & editing. **Katie Hampson:** Conceptualization, Funding acquisition, Supervision, Validation, Writing – review & editing. **Felix Lankester:** Conceptualization, Funding acquisition, Project administration, Supervision, Writing – review & editing. **Ahmed Lugelo:** Conceptualization, Data curation, Investigation, Project administration. **Joel Changalucha:** Conceptualization, Data curation, Investigation. **Kennedy Selestin Lushasi:** Conceptualization, Data curation, Investigation. **Anna Czupryna:** Conceptualization, Data curation, Investigation. **Emmanuel Mpolya:** Supervision, Writing – review & editing. **Katharina Kreppel:** Supervision, Writing – review & editing. **Sarah Cleaveland:** Conceptualization, Funding acquisition, Supervision, Writing – review & editing. **Sally Wyke:** Conceptualization, Formal analysis, Funding acquisition, Methodology, Supervision, Validation, Writing – review & editing.

## Declaration of Competing Interest

The authors declare the following financial interests/personal relationships which may be considered as potential competing interests:

Christian Tetteh DUAMOR reports financial support, equipment, drugs, or supplies, and travel were provided by Deltas Africa (Afrique One-ASPIRE). Felix Lankester reports financial support, administrative support, equipment, drugs, or supplies, and travel were provided by National Institute of Health. Katie Hampson reports financial support, equipment, drugs, or supplies, and travel were provided by 10.13039/100010269Wellcome Trust.

## Data Availability

All data on which discussion and conclusions are based are included in the manuscript.
